# Chemokine (C-C Motif) Ligand 2 (CCL2) in Sera of Patients with Type 1 Diabetes and Diabetic Complications

**DOI:** 10.1371/journal.pone.0017822

**Published:** 2011-04-12

**Authors:** Ruili Guan, Sharad Purohit, Hongjie Wang, Bruce Bode, John Chip Reed, R. Dennis Steed, Stephen W. Anderson, Leigh Steed, Diane Hopkins, Chun Xia, Jin-Xiong She

**Affiliations:** 1 Institute of Translational Medicine and School of Pharmaceutical Sciences, Nanjing University of Technology, Nanjing, Jiangsu, People's Republic of China; 2 Department of Microbiology and Immunology, College of Veterinary Medicine, China Agricultural University, Beijing, People's Republic of China; 3 Center for Biotechnology and Genomic Medicine, Medical College of Georgia, Augusta, Georgia, United States of America; 4 Department of Pathology, Medical College of Georgia, Augusta, Georgia, United States of America; 5 Atlanta Diabetes Associates, Atlanta, Georgia, United States of America; 6 Southeastern Endocrine and Diabetes, Atlanta, Georgia, United States of America; 7 Pediatric Endocrine Associates, Atlanta, Georgia, United States of America; University of Bremen, Germany

## Abstract

**Background:**

Chemokine (C-C motif) ligand 2 (CCL2), commonly known as monocyte chemoattractant protein-1 (MCP-1), has been implicated in the pathogenesis of many diseases characterized by monocytic infiltration. However, limited data have been reported on MCP-1 in type 1 diabetes (T1D) and the findings are inconclusive and inconsistent.

**Methods:**

In this study, MCP-1 was measured in the sera from 2,472 T1D patients and 2,654 healthy controls using a Luminex assay. The rs1024611 SNP in the promoter region of MCP-1 was genotyped for a subset of subjects (1764 T1D patients and 1323 controls) using the TaqMan-assay.

**Results:**

Subject age, sex or genotypes of MCP-1 rs1024611SNP did not have a major impact on serum MCP-1 levels in either healthy controls or patients. While hemoglobin A1c levels did not have a major influence on serum MCP-1 levels, the mean serum MCP-1 levels are significantly higher in patients with multiple complications (mean = 242 ng/ml) compared to patients without any complications (mean = 201 ng/ml) (p = 3.5×10^−6^). Furthermore, mean serum MCP-1 is higher in controls (mean = 261 ng/ml) than T1D patients (mean = 208 ng/ml) (p<10^−23^). More importantly, the frequency of subjects with extremely high levels (>99^th^ percentile of patients or 955 ng/ml) of serum MCP-1 is significantly lower in the T1D group compared to the control group (odds ratio = 0.11, p<10^−33^).

**Conclusion:**

MCP-1 may have a dual role in T1D and its complications. While very high levels of serum MCP-1 may be protective against the development of T1D, complications are associated with higher serum MCP-1 levels within the T1D group.

## Introduction

Chemokine (C-C motif) ligand 2 (CCL2) is commonly known as monocyte chemoattractant protein-1 (MCP-1). It belongs to a family of secreted proteins involved in immunoregulatory and inflammatory processes. MCP-1 is structurally related to the CXC subfamily of cytokines, which are characterized by two cysteines separated by a single amino acid. This protein displays chemotactic activity for monocytes and basophils but not for neutrophils or eosinophils. It binds to chemokine receptors CCR2 and CCR4.

MCP-1 is widely known as a pro-inflammatory cytokine due to its chemotactic activity. It has been implicated in the pathogenesis of many diseases characterized by monocytic infiltration, such as psoriasis, rheumatoid arthritis and atherosclerosis. Elevated MCP-1 serum levels have been reported in many diseases including coronary artery disease (CAD), hepatitis, obesity, acute myeloid leukemia and autoimmune diseases such as rheumatoid arthritis, chronic autoimmune thyroiditis [1]–[12].

Although it is less well appreciated, MCP-1 is also known to have anti-inflammatory properties. MCP-1 can stimulate the production of IL-4 [13], which is the primary Th-2 cytokine. Therefore, MCP-1 may play a protective role against some autoimmune diseases. Consistent with its anti-inflammatory activity, it was reported that serum MCP-1 levels were decreased in multiple sclerosis patients compared to healthy controls [14].

Limited data have been reported on MCP-1 in type 1 diabetes (T1D) [15]–[18], an autoimmune disease characterized by lymphocyte infiltration into the pancreatic islets and destruction of the insulin-producing islet β-cells in children and young adults. The findings range from significantly higher MCP-1 in T1D patients than controls [17], to no significant difference between T1D and controls [15], to significantly lower MCP-1 in pre-diabetic children with islet autoantibodies [18]. Due to the extremely small sample sizes (15–96 subjects per study group) in these studies, the results are, at best, inconclusive and inconsistent. Therefore, it is still unclear what role, if any, MCP-1 may play in the pathogenesis of T1D and its associated complications.

This study with 2472 T1D patients and 2654 controls was designed to have sufficient statistical power to answer three main questions related to the potential role of MCP-1 in T1D and its complications. 1) Is there a significant difference between T1D patients and healthy controls in serum MCP-1levels? 2) Do MCP-1 levels in T1D patients differ according to sex, age, duration of diabetes, metabolic control as measured by hemoglobin A1c and the presence of different diabetic complications? 3) Are serum MCP-1 levels genetically determined by a functional polymorphism in the MCP-1 promoter region?

## Results

### Serum MCP-1 levels in T1D patients and controls

**Figure 1:**
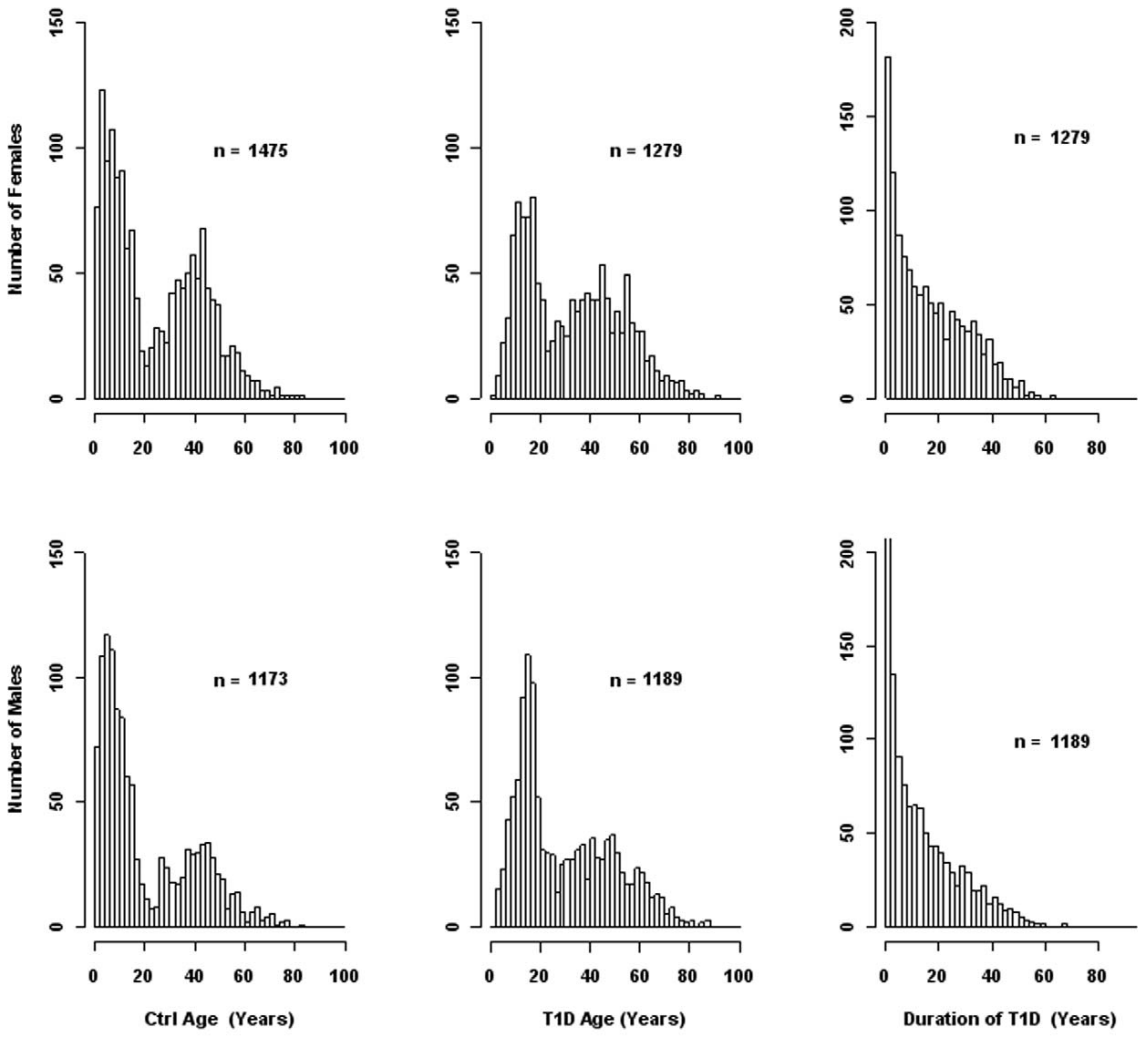
Summary of demographic information on study subjects.

**Figure 2:**
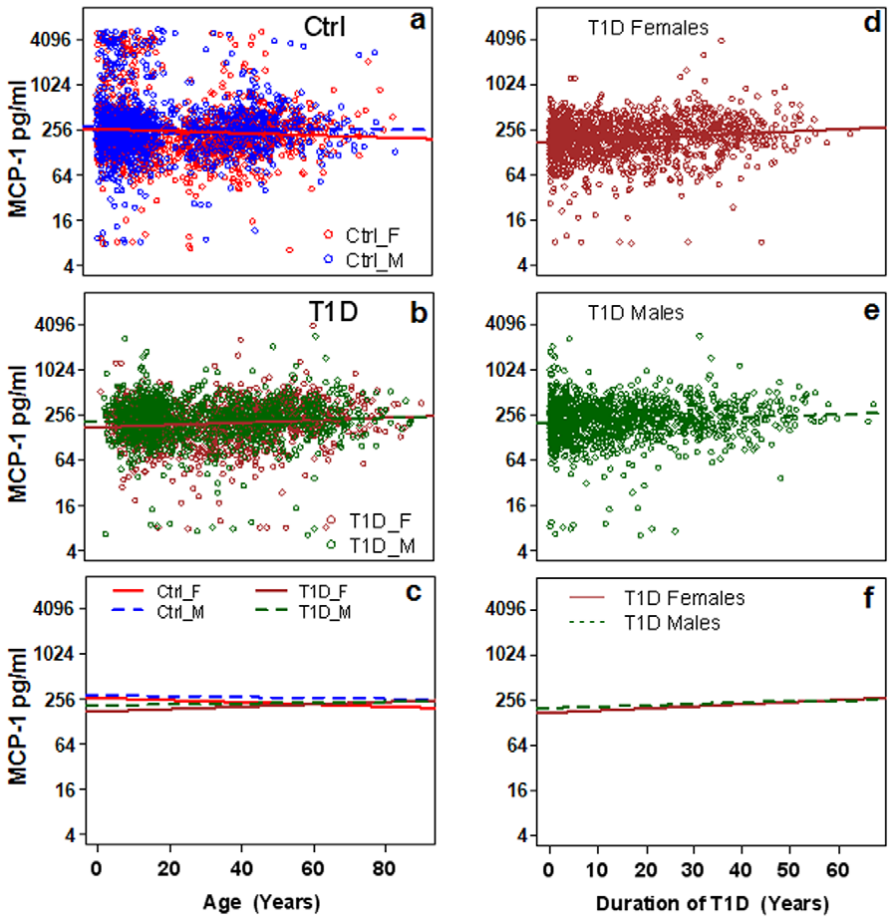
Regression graphs of the relationship between serum levels of MCP-1 relative to age of subjects (a, b & c) and duration of T1D (d, e & f). a) Relative levels of MCP-1 as a function of Age in females (β = −0.005, R = −0.06) and males (β = −0.0013, R = −0.02) in the control group. b) Relative expression of MCP-1 in serum of T1D females (β = 0.005, R = 0.11) and males (β = 0.002, R = 0.04). c) Mean serum MCP-1 levels (regression lines) according to age. d) Regression for duration of disease for female patients (β = 0.009, R = 0.14). e) Regression for duration of disease for male patients (β = 0.005, R = 0.08). f) Mean serum MCP-1 levels (regression lines) according to duration of T1D. β: slope of regression, R: correlation coefficient.

Serum MCP-1 levels were measured using a Luminex assay for 2472 T1D patients and 2654 control subjects. The sex and age distributions of the samples are presented in Figure 1. Regression analyses of the controls using MCP-1 as dependent variable and age as covariate suggested that MCP-1 levels did not differ according to age or sex of the control subjects (Figure 2a). Regression of the T1D patients indicated that MCP-1 levels did not differ according to age (Figure 2b), sex (Figure 2b), the duration of diabetes (Figure 2d, e and f) or hemoglobin A1c levels (data not shown).

### T1D patients have lower mean MCP-1 than controls

**Figure 3:**
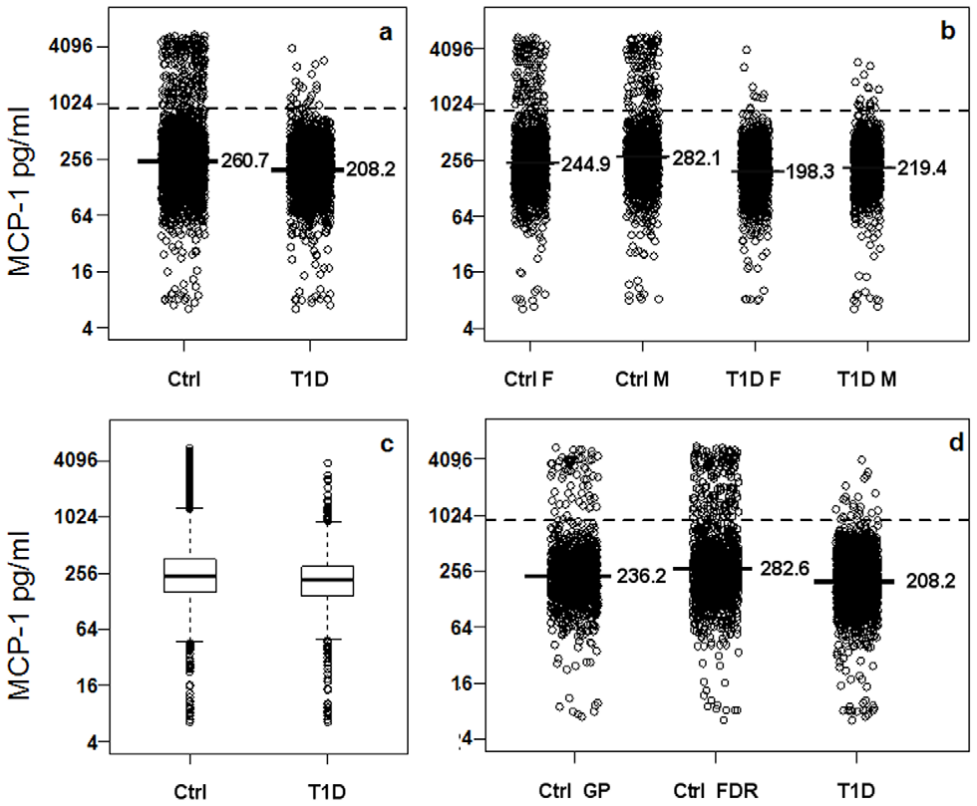
Serum MCP-1 differences between T1D and control groups. Dash lines mark the 99^th^ percentile value in controls. Solid lines and values on the right side represent the mean values of serum MCP-1 in the respective groups. A. Comparison between all T1D and all control subjects. b. Box plots for T1D and control groups. c. The control group is separated into first degree relatives of T1D (FDR) and general population (GP). d. Female and male subjects are separately plotted for both T1D and control groups.

Interestingly, the mean MCP-1 levels were slightly but significantly lower in the T1D group (mean = 208 ng/ml) compared with the control group (mean = 261 ng/ml) (p<10^−23^, Figure 3a). The differences between T1D and control subjects are highly significant in both male (p<10^−13^) and female groups (p<10^−13^) (Figure 3b). Box-plots for the MCP-1 in T1D and control groups (Figure 3c) also indicated that the 25^th^ and 75^th^ percentiles of MCP-1 levels differ between T1D and control subjects. Most importantly, a significantly higher percentage of control subjects (8.9%) had very high MCP-1 levels (defined as above the 99^th^ percentile value in patients, 955 ng/ml) compared to 1% of the T1D subjects (odds ratio = 0.11, p<10^−33^).

Since the control group contains two subsets of subjects, 1190 from the general population (GP) and 1464 first degree relatives (FDR) of T1D patients, we further examined whether the serum MCP-1 levels differ between these two groups. As shown in Figure 3d, the MCP-1 levels are slightly higher in the FDR (mean = 282 ng/ml) than GP controls (mean = 236 ng/ml) and both groups are significantly higher than T1D patients (p<10^−20^ and 10^−9^, respectively).

### MCP-1 levels are higher in T1D patients with complications

MCP-1 levels were further examined in T1D patients with different diabetic complications. With the exception of two complications (foot ulcer and autonomic neuropathy) that have smaller numbers of subjects, the mean levels of serum MCP-1are significantly higher in patients with a diabetic complication including hypertension, CAD, nephropathy, retinopathy, photocoagulation, blindness, dyslipidemia, amputation, and peripheral neuropathy than T1D patients without the respective complication (Table 1). Furthermore, patients with three or more complications have higher mean MCP-1 levels than patients with 0–2 complications.

**Table 1:** Serum MCP-1 levels (Log2) in T1D patients with diabetic complications.

Complications	Subject (n)	Log 2 Mean	Log2 SD	Ratio*	p-value
No complications	1740	7.65	0.92	–	–
Hypertension	326	7.89	0.93	1.18	0.0023
CAD	119	7.96	0.84	1.24	0.0093
Nephropathy	115	7.86	0.83	1.16	0.029
Retinopathy	302	7.90	0.89	1.19	0.00019
Photocoagulation	212	7.92	0.83	1.21	0.00019
Blindness	48	8.07	0.69	1.34	0.00010
Dyslipidemia	419	7.81	1.03	1.12	0.011
Amputation	15	8.09	0.68	1.36	0.050
Foot Ulcer	32	7.76	1.14	1.08	ns
Peripheral Neuropathy	221	7.94	0.80	1.22	3.0E-07
Autonomic Neuropathy	66	7.71	0.75	1.04	ns
Any complications	732	7.80	0.94	1.11	0.00018
Any one complication	273	7.69	0.94	1.03	ns
Any 2 complications	158	7.79	1.04	1.10	0.07
Any 3 or 3+ complications	301	7.92	0.87	1.21	3.5E-06

*Ratio was calculated for each specific category of complication in comparison to the group without any complication.

### rs1024611 polymorphism and serum MCP-1 levels

**Figure 4:**
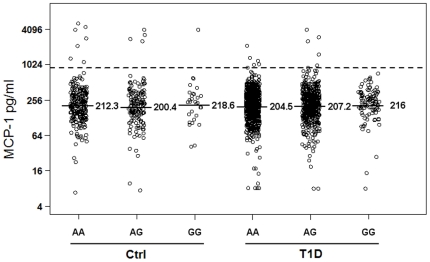
Serum MCP-1 values are plotted for each subject based on their −2518A/G SNP genotypes. Means for each group are marked by the solid lines and the values on the right side.

A single nucleotide polymorphism (SNP) is located at the −2518 position of the MCP-1 promoter region (rs1024611), which is genetically associated with several autoimmune diseases [19]–[21]. The SNP genotypes are also associated with MCP-1 gene expression in human pancreatic islets [22]. In heterozygous individuals for the rs1024611 SNP, allele-specific transcription of MCP-1 has been reported for human peripheral blood mononuclear cells treated with the inflammatory cytokine IL-1β [23]. Furthermore, the promoter polymorphism was correlated with increased serum MCP-1 levels in severe acute pancreatitis [24]. Therefore, the SNP was genotyped for 1764 T1D patients and 1323 GP controls to assess a potential genetic association between T1D and the SNP. The genotypic and allelic frequencies for this SNP are similar in T1D patients and controls (Table 2), suggesting that the MCP-1 SNP is not associated with T1D. Furthermore, the potential association between serum MCP-1 levels and the promoter SNP genotypes was assessed in the 1384 T1D patients and 702 controls with both genotype and serum protein data. As shown in Figure 4, the mean MCP-1 levels do not differ in the three different MCP-1 genotypes in either controls or T1D patients (p>0.1).

**Table 2:** Genotypic and allelic frequencies of MCP-1 SNP.

	T1D patients(n = 1764)	AbN controls(n = 1323)
Genotypes		
AA	952 (54.0%)	706 (53.4%)
AG	683 (38.7%)	507 (38.3%)
GG	129 (7.3%)	110 (8.3%)
Alleles		
A	2587 (73.3%)	1919 (72.5%)
G	941 (26.7%)	727 (27.5%)

## Discussion

Serum MCP-1 has been measured in T1D patients with or without diabetic complications as well as controls in several previous studies [15]–[18]. However, all these studies had extremely small sample sizes ranging from 15 to 96 subjects in each group and the conclusions from these studies were inconclusive and inconsistent. In this study, we measured serum MCP-1 in 2472 T1D patients in different age groups and with/without diabetic complications. The sample size is at least 25 times bigger than the largest study previously reported on MCP-1. This large dataset allowed us to examine the various parameters that may influence serum MCP-1 levels in T1D patients. Our study highlights the need for large dataset to detect small differences in the means for subject groups with different health status due to the large variation in the patient populations. Our results suggested that sex, age, hemoglobin A1c or duration of diabetes did not have a major impact on serum MCP-1 in T1D patients. However, we were not able to examine the relationship between MCP-1 and C-peptide levels because such data were not available on most subjects. Interestingly, the mean serum MCP-1 levels are 20–40% higher in T1D patients with most diabetic complications including hypertension, CAD, nephropathy, retinopathy, photocoagulation, blindness, dyslipidemia, amputation, and peripheral neuropathy. These observations are consistent with the general belief that MCP-1, a potent monocytes attractant, is a proinflammatory chemokine. Inflammation is a major hallmark of diabetic patients. Our study suggests that MCP-1 is increased in diabetic patients with complications, especially those with severe or multiple complications. It is therefore possible that reducing inflammation may help improve the health status of diabetic patients. It has indeed been found that diabetes induces kidney production of MCP-1 and that MCP-1 may be an important therapeutic target for diabetic nephropathy [25]–[28]. MCP-1 levels was also reported to be elevated in diabetic patients with coronary artery disease [29], [30]. Our results suggest that MCP-1 may also be involved in other complications related to diabetes.

Interestingly, the mean of serum MCP-1 level is slightly but significantly lower in T1D patients in comparison with healthy controls. These findings suggest that MCP-1 may play different roles in the development of T1D versus diabetic complications. Because MCP-1 is higher in controls than T1D patients, our data may suggest that MCP-1 may be protective against the development of T1D. This hypothesis is consistent with a previous study that reported significantly lower serum MCP-1 levels in prediabetic children marked by the presence of islet cell autoantibodies [18] . A protective role of MCP-1 against T1D is plausible based on the function of the molecule. It has been shown that no inflammatory infiltrates were detected in transgenic mice with the MCP-1 transgene under the control of the mouse mammary tumor virus long terminal repeat (MMTV-LTR) despite expression of high levels of biologically active MCP-1 in several organs and the serum [31]. One explanation of these findings is that high levels of MCP-1 in the vasculature can desensitize MCP-1 receptors on circulating monocytes. This hypothesis is consistent with our finding that the frequency of individuals with very high serum MCP-1 (above the 99^th^ percentile, Log2 = 9.9) is significantly higher in controls (8.9%) than in the T1D group (1%) (p<10^−33^). A second explanation of the findings is that high serum concentration of MCP-1 may simply cancel the chemoattractant gradient from the pathological site. A third explanation is that MCP-1 can stimulate the production of IL-4, which is the primary Th-2 cytokine, and has anti- inflammatory property through control of T cell polarization to Th2 responses [13]. The functional polarization of T cell subsets of lymphocytes plays a pivotal role in promoting or conferring risk to T1D in both humans and animal models [32]. An increase in the expression of Th1 cytokines and a corresponding decline in the production of Th2 cytokines (IL-4) is correlated with recent-onset T1D [33]. Predominance of high Th1 cytokines is a main factor to cause destruction of pancreatic β-cells. In contrast, a constitutive release of IL-4 from pancreatic β-cells is able to prevent insulitis development in NOD-IL-4 transgenic mice [34]. Immune-intervention strategies have been explored to establish a good environment that is attributed to a change in the set of autoreactive T cells from their Th1to the protective Th2 phenotype. MCP-1 may have a dual role in the pathogenesis of T1D. On one hand, MCP-1's pro-inflammatory activity of monocyte chemoattraction may contribute to leukocyte infiltration to the pancreatic islets [31]. On the other hand, very high levels of serum MCP-1 may exert protective effect against the development of T1D through one or more mechanisms discussed above.

Finally, the lower MCP-1 observed in T1D patients than controls may simply reflect the decline of IL-4 during or after T1D development. IL-4 can regulate and stimulate expression of the MCP-1 by human endothelial cells [35]. Studies in human and animal models suggested that IL-4 production by PBMC or T cells was significantly lower in new-onset T1D patients than the controls [33]. Therefore, the contribution of MCP-1 to T1D pathogenesis may depend on its expression levels and the overall immunological milieu in the at-risk subject. To fully understand its role in T1D pathogenesis, it must be studied in the context of other immune molecules including IL-4 and other Th1 and Th2 cytokines and chemokines. Furthermore, the possible protective role of very high levels of serum MCP-1 against the development of T1D must be tested in prediabetic subjects and in a large prospective cohort.

## Methods

### Ethics statement

This study was approved by the Medical College of Georgia Institutional review Board. All patients provided written informed consent for participation in the study and donation of samples.

### Patient population

Serum MCP-1 was measured in 2, 472 patients with T1D and 2,654 healthy controls, which consist of 1,464 FDR subjects and 1,190 subjects from the general population (GP). All subjects were recruited in Georgia, USA, mainly in the Atlanta and Augusta areas. Autoantibodies were only measured in a subset of patients and controls. C-peptide data were not available for the subjects. The sex and age distributions for all subjects of and duration of diabetes for the patients were summarized in Figure 1. A total of 1764 T1D patients and 1323 GP controls were also genotyped for the SNP rs1024611 located at nucleotide −2518 of the MCP-1 promoter.

Blood was drawn from all subjects and was allowed to clot at room temperature for 30 minutes before centrifugation at 3,000 g at 4°C for 10 minutes. Then blood samples for serum were stored in −80 freezers until use. All samples were retrospectively analyzed. In the experiment, each sample was aliquoted into wells of 96 well v-bottom plates and each plate contained similar numbers of samples from T1D patients and controls. From blood collection to the assay, none of the samples had more than three freeze/thaw cycles and each sample usually had only one freeze/thaw cycle.

### Luminex assay for MCP-1

MCP-1 in serum was measured using a Luminex assay from RnD Systems (RnD Systems, Minneapolis, MN, USA) according to manufacturer's protocol. Briefly, the kit is based on sandwich immuno-assay, which consists of dyed microspheres conjugated with a specific monoclonal capture antibody. Serum samples were incubated with the antibody-coupled microspheres after they were incubated with biotinylated detection antibody before the addition of streptavidin-phycoerythrin. The captured bead-complexes were then read by a Bioplex 100 system (Biorad Laboratories, Hercules, CA, USA) with the following instrument settings: events/bead: 50, minimum events: 0, Flow rate: 60 ul/min, Sample size: 50 ul, discriminator gate: 12800. For all the assays median fluorescence intensity (MFI) were collected.

MCP-1 concentration (pg/ml) for each subject was estimated using a linear regression fit to the standard curve of recombinant MCP-1 protein standards included on each plate using a 3-fold serial dilution series. The log of the observed median fluorescence intensity (MFI) for the dilution series samples were regressed on the log of the known concentration for these samples. The concentration of subject samples with an MFI that was either above the largest MFI or below the smallest MFI of the known standards were set to the estimated concentration for the appropriate extreme known standards. Samples with a CV among microspheres within the well that was greater than 100 were removed since the MFI for such wells are unreliable. The estimated MCP-1 concentrations were on the log scale, and as such, all analyses were conducted using ln(MCP-1 concentration). Estimation of MCP-1 concentration using standard curves were conducted using R [36].

### Statistical analysis for serum MCP-1 protein

The potential difference between T1D patients, healthy controls and other subsets of subjects was initially examined using a t-test. The potential association of MCP-1 levels with age, hemoglobin A1c levels and duration with diabetes was examined using linear regression. Male and female subjects were analyzed separately and also jointly analyzed by including sex as a co-variate. For statistical analysis, R was used for statistical computing and graphics in the present study [36].

### SNP genotyping and data analysis

Genotyping of MCP-1 rs1024611 SNP was carried out using the TaqMan-assay as described previously [37]. The assay was designed and validated by Applied Biosystems. Amplification reactions were performed in a 5 ul final volume in optical 384-well plates. PCR was carried out with 2 min at 50°C, 10 min at 95°C followed by 40 cycles of 15 s at 95°C and 1 min at 60°C using an ABI9700 real-time PCR system (Applied Biosystems). The frequency of alleles and genotypes in the patient subgroups and normal controls were compared using x^2^ test.
